# Awareness of ethical issues in medical education: an interactive teach-the-teacher course 

**DOI:** 10.3205/zma001044

**Published:** 2016-05-17

**Authors:** Costanza Chiapponi, Konstantinos Dimitriadis, Gülümser Özgül, Robert G. Siebeck, Matthias Siebeck

**Affiliations:** 1 Hospital of the University of Magdeburg (OvGU), Department of General, Visceral and Vascular Surgery, Magdeburg, Germany; 2 Hospital of the University of Munich (LMU), Department of Neurology, Munich, Germany; 3 Hospital of Aalen, Department of Gynaecology, Aalen, Germany; 4 Munich School of Philosophy, Munich, Germany; 5 Hospital of the University of Munich (LMU), Department of General, Visceral, Vascular and Transplantation Surgery, Munich, Germany

**Keywords:** ethics, medical education, moral decision-making, culturally learned patterns

## Abstract

**Purpose:** We conducted an international, interdisciplinary teach-the-teacher course to sensitize physicians from different countries to ethical issues in medical education. The purpose of this study was to assess the effects of this course.

**Method: **Before and after participating in a short session on ethical issues in medical education, 97 physicians from different countries in Africa, Asia, and Europe completed a self-assessment questionnaire on their competence and interest in this field. The short session consisted of working in small groups to identify, analyze and discuss ethical dilemmas described in case vignettes adapted from published examples or written by medical students. In addition to the questionnaire, we conducted a large-group experience to explore four basic orientations of participants in ethical thinking: relativism, intentionalism, consequentialism, and absolutism.

**Results: **We found a significant self-perceived increase in the participants’ ability to identify and describe ethical issues and students’ dilemmas, in their knowledge about these issues and teaching professionalism, and in their ability to describe both students’ perspectives and teachers’ and students’ behaviors. In addition, participants’ feeling of understanding their own culturally learned patterns of determining what is right and wrong increased after taking part in the course. The four contrasting basic ethical orientations showed no significant differences between participants regarding nationality, age, or gender.

**Conclusion: **Ethics of education is an important issue for medical teachers. Teachers’ self-perceived competence can be increased by working on case vignettes in small groups.

## Zusammenfassung

**Zielsetzung: **Wir haben einen internationalen, interdisziplinären teach-the-teacher Kurs durchgeführt mit dem Ziel, Ärzte unterschiedlicher Nationalitäten für ethische Probleme in der medizinischen Ausbildung zu sensibilisieren. Das Ziel der vorgestellten Arbeit war, die Effekte dieses Kurses auf die Teilnehmer zu untersuchen.

**Methode:** Vor und nach dem Kurs beantworteten 97 Ärzte aus Afrika, Asien und Europa einen Selbsteinschätzungsbogen bezüglich ihrer Kompetenz und ihrer Interessen zum Thema ethische Probleme in der Medizindidaktik. Der Kurs bestand aus einer Kleingruppenübung mit dem Ziel, ethische Dilemmata in Fallvignetten zu identifizieren, analysieren und diskutieren. Diese Fallvignetten wurden aus bereits publizierten Beispielen in modifizierter Form übernommen oder von Medizinstudenten neu verfasst. Zudem wurde eine Großgruppenübung durchgeführt, um die Zugehörigkeit der Teilnehmer an einer der vier kontrastierenden ethischen Grundorientierungen zu analysieren (Relativismus, Intentionalismus, Konsequentialismus und Absolutismus).

**Ergebnisse: **Es kam zu einem signifikanten Anstieg der selbst eingeschätzten Fähigkeit der Teilnehmer, ethische Probleme zu identifizieren und zu beschreiben. Das Wissen der Teilnehmer über die Probleme und über das Unterrichten von Professionalität und deren Fähigkeit, nicht nur die studentischen Perspektive, sondern auch das Verhalten von Lehrer und Lernenden, zu beschreiben, konnte ebenfalls gesteigert werden. Zudem stieg nach der Teilnahme am Kurs das Gefühl der Teilnehmer, die eigenen kulturbedingten Muster der Beurteilung von Richtig und Falsch besser zu verstehen. Es konnte kein landes-, alters- oder geschlechtsspezifischer Unterschied in den vier verschiedenen ethischen Orientierungsrichtungen nachgewiesen werden.

**Schlussfolgerungen: **Ethik der Ausbildung ist ein wichtiges Thema für didaktisch tätige Ärzte. Die selbst eingeschätzte Kompetenz kann durch den beschriebenen interaktiven Kurs gesteigert werden.

## 1. Introduction

Medical education aims to ensure that every student acquires not only the knowledge and skills required to treat patients adequately (and thus ethically) but also a professional identity “so that he or she comes to think, act, and feel like a physician” [1], [2]. In the last few years, a lot has been written about the need for teaching and reflecting on professionalism [3], [4], [5], [6], [7], [8] in order to assist learners in developing their own professional identity. According to Doukas [9], a good physician should act scientifically and in a clinically competent way by practicing according to the best evidence, keep the patient’s health-related interests in mind and pass on medicine to future physicians. 

The German NKLM (Nationaler Kompetenzbasierter Lernzielkatalog Medizin, http://www.nklm.de) is a newly developed catalogue of competencies that medical students should acquire during their education and describes the physician’s “roles” [10]. One of these roles is that of a “scholar,” “a lifelong learner,” who is responsible also for educating patients, institutions, politicians, and medical students (§6.3) [11]. Towards this aim, the physician is supposed to know about several different teaching, learning, and examination methods (§6.3.1.1) and be capable of reflecting on and evaluating teachable moments (§6.3.1.1-2). Reiser [12] wrote that “The patient is shielded by a wall of ethical values articulated and expanded for over two millennia,” pointing out the need for ethical principles in the teacher-student relationship. In recent years, this relationship has moved into the focus of medical didactic research [13], [14], [15].

At some point, it became clear that medical teachers must learn to mediate between their patients and students. Both have rights that the teacher needs to respect and that patients and students need to respect when they interact. Patients’ and students’ interests might sometimes conflict [16], [17], [18], [19], [20]. Therefore, it is a core task of clinical teachers to assess the risk of students’ participation in medical care, supervise students and educate patients about the benefits of student participation in their care and about their own role in helping to train the next generation of physicians [21]. This core task is reflected in another recent catalogue of core competencies for medical teachers (the “KLM”) developed in Germany; this catalogue includes fields such as “educational action in medicine,” “role modelling and professionalism,” and “reflection and advancement of personal teaching practice and systems related teaching and learning” [22]. 

For several years, the Ludwig Maximilian University, Munich, Germany, has been performing teach-the-teacher courses in cooperation with several foreign universities. The main subjects included problem-based learning (PBL), oral and written examinations, seminars, and objective structured clinical examination (OSCE). When the University of Jimma, Ethiopia, expressed the wish for a teach-the-teacher course on the “ethics of medical education,” we designed a new course and subsequently offered it on four occasions to 97 physicians from Africa, Asia, and Europe. The course aims to assist participants in refining their moral thinking and developing a methodology for their own moral decision-making. 

In developing the course, we concentrated on five aspects:

1. The learning unit must build on the participants’ own experience.
2. It should emphasize the need to teach professionalism.
3. It should link with what educators usually teach.
4. It must be assessable.
5. The teaching materials need to be readily understandable and relevant for the learner in order to promote this field in an international context.

In 1994, Reiser [12] suggested to hold case discussions periodically in which “students could raise ethical dilemmas of their clinical work (…) or association with instructors.” Therefore, we decided to use a similar method and ask medical students to identify dilemmas in their educational experience that should be discussed by instructors in the PBL session. 

This study was not concerned with how to teach medical students ethical thinking for the medical care of patients, but evaluated whether our teach-the-teacher course had a positive effect on medical educators’ awareness of ethical issues. The study used self-assessment questionnaires to evaluate how the course affected medical teachers’ ethical thinking concerning their daily interactions with both patients (as physicians) and students (as instructors). The study also explored four basic orientations of participants’ ethical thinking (relativism, intentionalism, consequentialism, and absolutism) to examine whether these showed cultural differences.

## 2. Methods

We introduced a novel, international 3-step 4-hour course that included a small face-to-face lecture on definitions and core issues in medical ethics; PBL, consisting of small-group discussions of short case vignettes; and a large-group exercise that aimed to assess the participants’ basic orientations in ethical thinking. The course was designed and held by the authors of this manuscript four times between December 2010 and December 2011 with a total of 97 voluntary participants from Africa, Asia, and Europe.

In the short lecture on ethics, participants were given a definition of ethics in medical education as a collection of adequate behaviors of practitioners in order to balance their interaction with students and patients. Participants were asked to bring their own experience and beliefs into the discussion, which took place in English, since the participants came from different countries.

We chose to use case vignettes because when we asked participants at the beginning of the course for examples of ethical issues they had faced in medical education, many had difficulties in identifying any such issues. Some case vignettes were modified from published examples to make them suitable for the PBL technique [23], [24], [25], [26], and some were newly written specifically for the course on the basis of discussions with medical students. The cases were selected to provide a framework for identifying dilemmas commonly encountered while working as a medical educator. Examples of dilemmas incorporated into the vignettes are as follows: lack of adequate supervision of students and young doctors [21]; lack of adequate information given to patients about procedures being performed by students [21], [27]; avoidance (sending a younger colleague to deal with a more distressing issue); manipulation (educators misusing their power); and lack of empathy and avoidance of “difficult patients.” These cases were discussed in small groups of 10-12 participants. 

Finally, we used a slightly modified version of the method described by Pedersen [28] as a large-group experience to explore four basic orientations of participants in ethical thinking: relativism, intentionalism, consequentialism, and absolutism. 

Before and after the course, all participants completed self-assessment questionnaires to evaluate the effects of the course on their interests and perceptions regarding ethical issues of medical education. Because we were unable to find a suitable assessment instrument in the literature, we created a 12-item questionnaire (see Table 1 (Tab. 1)).

Participants rated each item on a 6-level bipolar anchor scale ranging from 1 (completely disagree) to 6 (completely agree). We did not assess the reliability or validity of the newly created instrument. In addition, we performed a feedback discussion, during which we gathered qualitative data. 

### 2.1. Data analysis

We calculated mean values for the questionnaire responses and used a paired t test to compare responses before and after the teaching course. P<0.05 was considered statistically significant; we made no adjustment for multiple testing. 

## 3. Results

**Figure 1:**
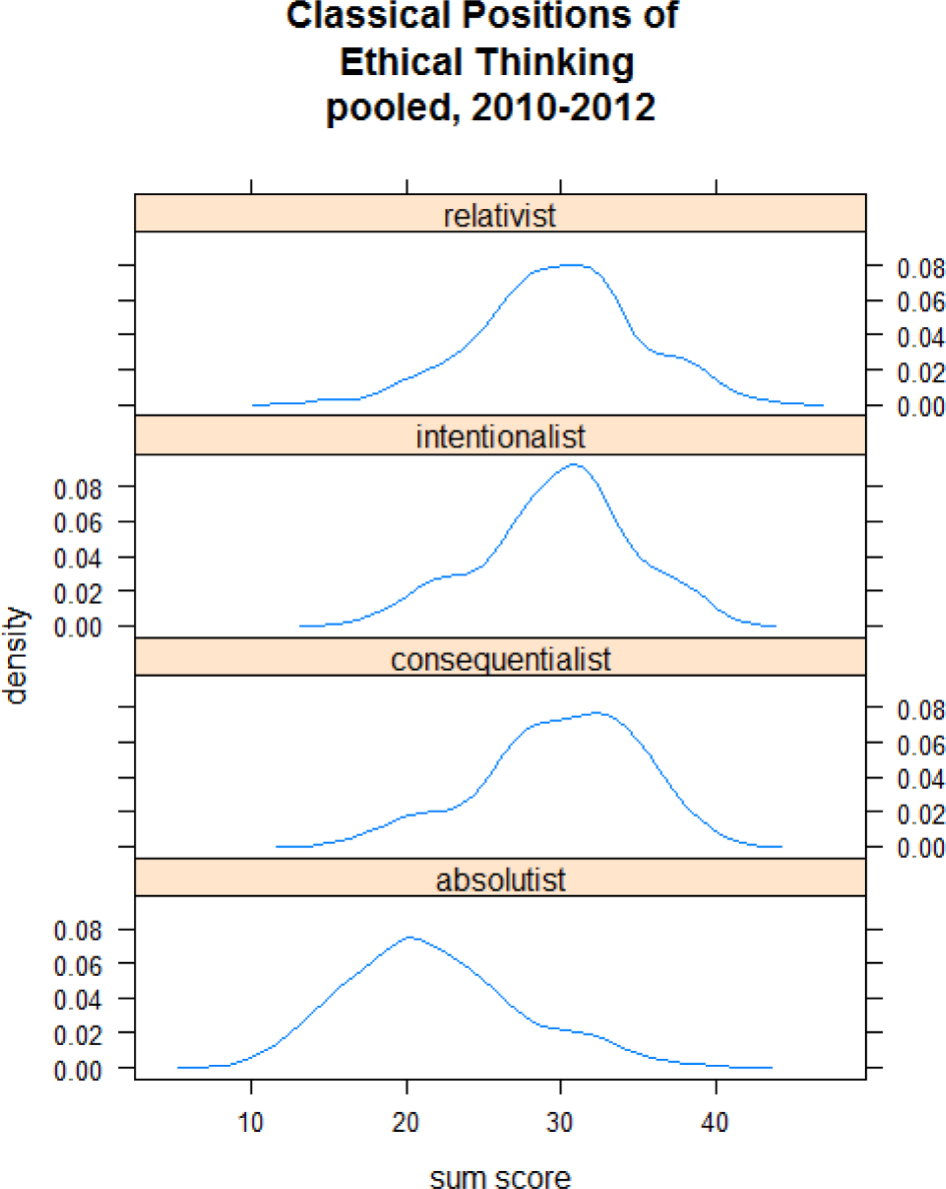
Participants’ ratings (n=97) on the 4 classical positions of ethical thinking according to Pedersen [28]

The median age of the 97 participants was 32 years (range: 21 to 76); 26 were female and 71 male. The participants’ nationality was Ethiopian (60); Polish (13), German (5), or other (19). Figure 1 (Fig. 1) summarizes the results of the four ethical orientation test by Pedersen [28]. Affiliation with the absolutist’s view was relatively low compared to the other scales. Analysis of the four different orientations in ethical orientation revealed no significant effect of gender, age, or nationality (see Figure 1 (Fig. 1)).

**Figure 2:**
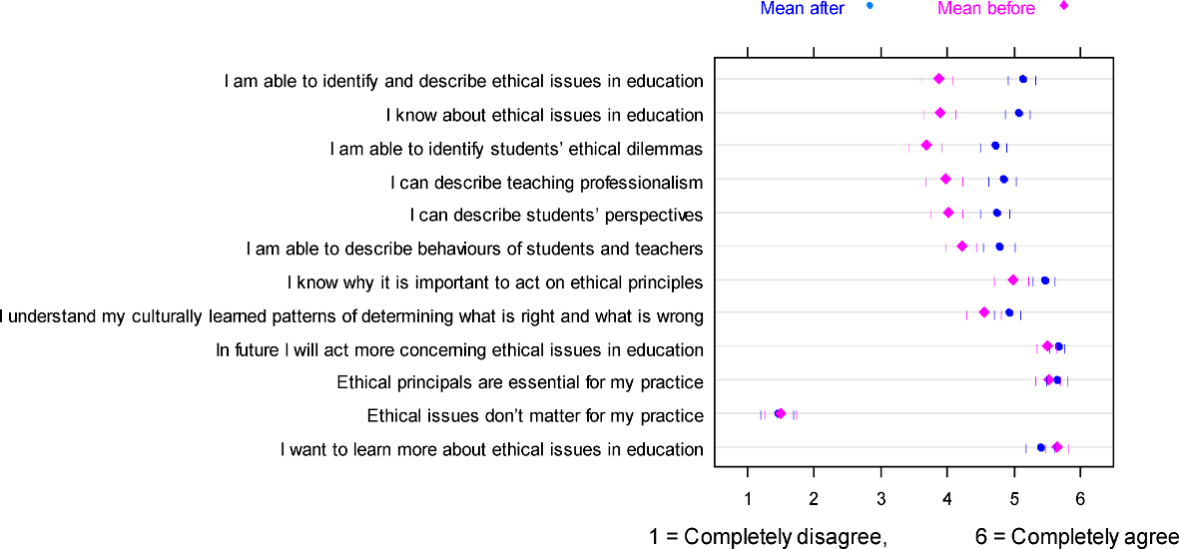
Results of the self-assessment questionnaire (n=90) on ethical issues in medical education performed at the beginning and end of the session. Symbols represent mean values, vertical bars denote the 95 % confidence interval. Items were sorted according to the size of change (difference before and after the session).

Ninety pairs of the self-assessment questionnaires (before and after the course) were completed and returned. After completing the course, we found a significant increase (p<.05, no adjustment for multiple testing) compared to baseline in the following 8 items (see Table 1 (Tab. 1)): Items 1, 2, 3, 4, 5, 6, 7, 11. The score for the remaining 4 items showed no significant change: Respondents agreed strongly with items 9, 10, and 12 (mean [SD] scores on the 6-point scale: 5.7 [0.9], 5.5 [0.9], and 5.5 [0.7]) both before and after the course and disagreed strongly with item 8 (mean [SD]: 1.5 [1.1]). Figure 2 (Fig. 2) summarizes the results of the self-assessment questionnaire performed at the beginning and end of the course. 

## 4. Discussion

To our knowledge, our course is the first international teach-the-teacher course on the ethics of teaching medicine. The aim of the course is to enable medical teachers to recognize, reflect on and analyze ethical dilemmas in teaching medicine in an international context. In fact, there is “an inherent conflict within clinician educators as we balance the roles of healthcare provider to patients in need of care with that of educator of learners in need of teaching” [29]. 

Teacher training used to be mostly informal, and teaching medicine was predominantly experiential and context dependent. Because in most countries the principals of teaching medicine were not specifically taught at medical school, and a resident was expected to be able to teach interns and medical students, teach-the-teacher courses on teaching methods and skills were introduced. These courses foster the professionalization of medical teaching. Recently, in Germany a comprehensive national framework was developed to promote standards for formal faculty development programs across institutions [30]. However, to our knowledge the aim of the course described in this paper, i.e. to discuss dilemmas encountered while balancing the roles of healthcare provider and medical educator, is a novel approach. 

Some years ago, the University of Toronto, Canada, developed a curriculum based on lectures on the definitions, causes, and consequences of physician-patient sexual misconduct and teacher-learner mistreatment and harassment [31], [32]. The course included also a workshop, which was based on discussing case vignettes depicting crucial situations. Our course goes beyond that in that it targets medical educators from different countries and considers moral issues in medical education on an international basis. 

The first objective of the course was to increase participants’ awareness of the issues. The results of the self-assessment questionnaire showed that participants are aware of the importance of ethical dilemmas in education and are highly motivated to learn more about them. At the beginning of the course, however, several participants were unable to define ethical dilemmas in medical education, although they were able to define ethical dilemmas in the care of patients.

In a short lesson on ethics, participants were told about a definition of ethics of medical education as a collection of adequate behaviors of practitioners in order to balance their interaction with students and patients. The next part of the course consisted of PBL, which was chosen as an additional method because it better corresponds to the nature of the ethical enquiry itself [33]. The chosen vignettes depicted some classic ethical problems in medical education and addressed some of the 10 issues identified by Singh as unethical behaviors in education [34]: having inappropriate relationships with students (sexual, business partnerships, drinking etc.); violation of clearly stated college rules and educational procedures; failing to perform duties (no teaching, chaos, wrong attitude toward the teaching profession, etc.); imposing personal views on students unrelated to the subject of a lesson or promoting view that do not represent the main stream (extreme political or religious views, views on controversial social issues, interest of a particular social group, etc.); improper grading, partiality, and lack of fairness (based on who is liked, who is not, race, past performance, socioeconomic background, etc.); exposing students to embarrassment or disparagement (emotional or psychological harassment); invading student's privacy; engaging students in unethical behavior; accepting gifts and favors; deceiving students and their parents.

An unexpected and interesting finding was that the test by Pedersen [28] on orientation in ethical thinking found no nationality-, gender-, or age-specific differences. Although our sample was not large enough to generalize the results, it might suggest that the ethics of medical professionals is influenced more by their profession than by their nationality, gender, or age. This might in turn imply that medical education in different countries reaches a similar degree of ethical orientation among physicians, a sort of “hidden moral curriculum” [15].

At the end of the course, participants felt that they had significantly improved their awareness of ethical issues in medical education, indicated by the fact that scores increased significantly in 8 of the 12 items. The remaining 4 items showed no significant change after the course, but for these items participants had indicated high levels of agreement (3 items) or disagreement (1 item) already at the initial, pre-course assessment.

A limitation of this study is the use of self-assessment for evaluation. However, this course must be seen as a pilot intervention to raise sensibility of medical educators for ethical issues in an international context. As mentioned above, to our knowledge no similar teach-the-teacher course has been described before. Teachers are taught about learning and teaching methods and examination techniques but only rarely about the ethics of medical education. The newly designed German NKLM [http://www.nklm.de] is innovative in this respect in that it requires physicians to be able to reflect on and recognize the limits of teaching situations [10]. 

We chose the case vignettes without strong criteria and solely on the basis of our and the students’ opinions about potential ethical dilemmas in medical education, because we wanted to create an easy, well-understandable, learner-centered course. Because at the beginning of the course participants often had difficulties in defining and recognizing ethical dilemmas in medical education, we decided to offer them some examples as a base for their discussion.

Learning to recognize and address everyday ethical issues in medical education requires training and integration. A “moral imagination” must be developed and cultivated for this aim [35]. With this course, we wanted to initiate a new “moral imagination” in medical educators in an international context.

## 5. Outlook

Raising awareness for a problem is just a first step. Increasing the number of medical educators who know about ethical dilemmas they may face at work can help in several ways. First, this knowledge can help them to make better decisions in difficult situations. If educators are aware of the existence of different ethical positions, they will have more insight into what factors may play a role in decision-making. Second, educators’ awareness of such problems can help them to attract others’ attention to these issues. The question whether the knowledge gained in a course like ours can be transferred into everyday life situations, by changing behavior of the participants, would be an interesting subject for further research. 

## Acknowledgements

The authors wish to express their gratitude to the faculty of the College of Public Health and Medical Science College at Jimma University and their Dean, Prof Abraham Haileamlak, for generating the idea to teach ethics of education, helping to perform the course and improving the manuscript. They thank also Jacquie Klesing, Board-certified Editor in the Life Sciences (ELS), for editing assistance with the manuscript. Funding for the teacher training events was from Center for International Health at LMU, DAAD and BMZ. 

## Competing interests

The authors declare that they have no competing interests.

## Figures and Tables

**Table 1:**
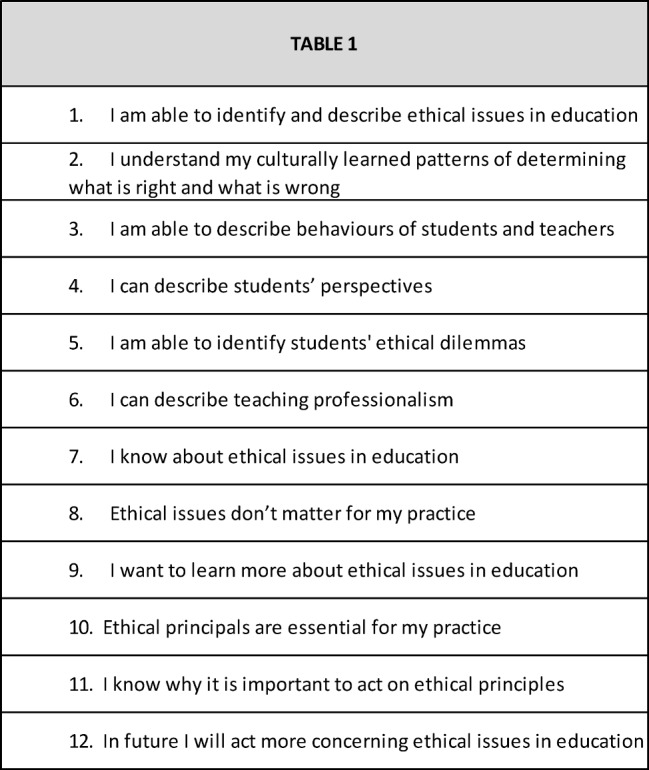
12-item questionnaire created for a teach-the-teacher course on the ethics of medical education. Participants completed the questionnaire at the beginning and end of the course.
